# Effect of 1-Week Weight Loss While Maintaining Total Body Water on Jump Performance

**DOI:** 10.1155/2024/6458445

**Published:** 2024-10-24

**Authors:** Hiroyuki Sagayama, Makiko Toguchi, Jun Yasukata, Rie Tomiga-Takae, Yujiro Kose, Masahiro Ikenaga, Takaaki Komiyama, Mamiko Ichikawa, Nemanja Lakicevic, Yasuki Higaki, Hiroaki Tanaka, Hiroyuki Nunome

**Affiliations:** ^1^Institute of Health and Sport Sciences, University of Tsukuba, Tsukuba, Japan; ^2^Advanced Research Initiative for Human High Performance (ARIHHP), University of Tsukuba, Tsukuba, Japan; ^3^Japan Institute of Sports Sciences, Japan High Performance Sport Center, Tokyo, Japan; ^4^Center for General Education, Kagoshima University, Kagoshima, Japan; ^5^Graduate School of Sports and Health Science, Fukuoka University, Fukuoka, Japan; ^6^Department of Sports and Life Science, National Institute of Fitness and Sports in Kanoya, Kagoshima, Japan; ^7^Faculty of Engineering, Nishinippon Institute of Technology, Fukuoka, Japan; ^8^Center for Education in Liberal Arts and Sciences, Osaka University, Osaka, Japan; ^9^Institute of Sports Science and Medicine, Teikyo University, Tokyo, Japan; ^10^Department of Psychology of Education and Pedagogy, Moscow State University, Lomonosov, Russia; ^11^Faculty of Sports and Health Science, Fukuoka University, Fukuoka, Japan

**Keywords:** body composition changes, jumping ability, physical function, weight loss program

## Abstract

Jumping performance is influenced by body composition and excess fat mass impairs performance. Maintaining optimal fat mass and fat-free mass (FFM) is crucial for enhancing jump height. However, there is limited evidence on short-term weight loss programs that reduce fat mass without water restriction and their effects on muscle function and jumping performance. This study aimed to clarify the effects of a 1-week weight loss program on jumping height and muscle function of volleyball players. The weight loss group engaged in two 40 min slow-paced jogging sessions in addition to their daily training routine. Energy intake was restricted without limitations on water intake. Total body water and body composition using the deuterium dilution method, muscle strength, and jump height before and after 1 week were evaluated for those in the weight loss and control groups. Body mass was significantly reduced in the weight loss group (−2.7 ± 1.3%, *p* < 0.05) with a significant reduction in fat mass (−17.7 ± 10.7%, *p* < 0.05). Meanwhile, there were no significant changes in total body water or FFM. Muscle strength and power tests indicated no significant differences between the groups; no notable differences were observed in handgrip strength or knee extension torque. The height of a single vertical and continuous jump remained consistent pre- and postintervention in the control group. In the weight loss group, although the height of a single vertical jump exhibited a slight decline postintervention, the height of a continuous jump displayed no significant changes. The short-term weight loss program significantly reduced fat mass without compromising muscle function, which is crucial for sports performance. These findings may benefit other athletes who require fat mass reduction while maintaining muscle function and help create new programs during specific training phases.

## 1. Introduction

In volleyball, players are required to perform essential actions, such as serving, spiking, and blocking in the air. Jumping higher provides a big advantage for players, and their physique (height) and jumping capability have significant roles in their performance. Therefore, improving jumping performance is crucial for gaining an advantage in volleyball matches [1]. Body composition, particularly fat mass (FM) and fat-free mass (FFM), can significantly impact player jumping capabilities [2]. Previous research has indicated a negative correlation between the percentage of body fat and the resultant vertical jumping height, in which body fat accounts for a significant portion of the variability in jumping performance [3]. These findings suggest that excessive FM might compromise jumping performance and highlight the essential need for players to maintain an optimal body composition year-round. Thus, volleyball players seeking to enhance their vertical jump height should focus on reducing excess FM while maintaining or enhancing FFM and lower limb strength through training and dietary practices. However, the current literature lacks evidence on weight-loss programs specifically tailored for volleyball players to enhance their jumping capabilities.

Previous studies demonstrated that weight reduction through a ketogenic diet decreased FM and improved countermovement jumps among athletes [4]. Koral and Dosseville [5] reported no significant differences in squat jump, countermovement jump performance, and repetition of 5-s judo movements with a 2%–6% reduction in body weight in elite judo athletes over a short period. Fogelholm et al. [6] investigated two methods of weight reduction, comprising gradual and rapid, in male wrestlers and judo athletes and reported a 6%–8% increase in the height of vertical jumps with gradual weight reduction. However, regarding weight loss for weight-classified athletes, restrictions on water intake and dehydration may lead to a reduction in body water, thereby making these weight reduction methods impractical for athletes in other sports.

In our previous study, we developed a 1-week supervised weight loss program and investigated its impact on weight, body composition, and overall health [7]. This program combined high-volume, low-to-moderate intensity exercise with energy intake restriction [7, 8]. Participants in this program engaged in 1 h slow-paced jogging and other leisure activities conducted three times a day while consuming only the provided foods without water restriction. The results revealed a significant weight reduction (−3.5%), decrease in FM, and maintenance of FFM. In addition, a modified version of this program tailored for student-athletes demonstrated similar reductions in FM while preserving FFM [8]. If this weight loss program can reduce FM while maintaining FFM and lower limb strength, it could enhance the jumping capability of volleyball players. Therefore, in this study, we aimed to elucidate the impact of a 1-week weight loss program on jumping performance and muscle function of volleyball players. We hypothesized that implementing a 1-week weight loss program, which included energy intake restriction without water restriction, would reduce FM while maintaining FFM, muscle strength, and power. Consequently, the program would enhance jumping height performance in volleyball players.

## 2. Materials and Methods

### 2.1. Participants

Twenty-four athletes (20 males and 4 females) were divided into the weight loss group (WL) and control group (CON) (WL: age 20 ± 1 years; height 172 ± 6 cm; *n* = 12; CON: age 22 ± 1 years; height 172 ± 5 cm; *n* = 12), including 18 who underwent performance testing.  Table 1 lists the physical characteristics of the participants in each group.

All participants attended an informational meeting where they received detailed information about the study's benefits and potential risks. Written informed consent was obtained from all participants, and the Institutional Review Board of Fukuoka University (Approval No. 14-08-01) approved the protocol. The study adhered to the ethical guidelines outlined in the Declaration of Helsinki and the ethical standards and guidelines for sports medicine and exercise science research [9].

### 2.2. Protocol

The WL group weight loss period was set to 1 week, and body composition and exercise performance were measured before and after weight loss; the CON group underwent simultaneous measurements. Body composition was calculated using the stable isotope dilution technique before and after the intervention program. Participants were instructed to collect urine samples on days 0 and 7 (at baseline and 3 and 4 h after baseline for both days) to calculate their total body water (TBW). The TBW, FFM, and FM calculation methods and validation are described in our previous study [10, 11]. Participants in both groups were instructed to refrain from overeating and binge eating and sustain a stable body weight for ≥ 2 weeks preceding the measurements. The CON group was instructed to maintain regular daily food intake and physical activity levels during the study period. In addition to their daily sport-associated activities, the WL group engaged in slow jogging sessions lasting 40 min twice per day (in the early morning and after club activities) and aimed to expend approximately 300–400 kcal per session. During each meal, participants verified the completion of all training and jogging sessions throughout the intervention. The typical schedule during the intervention included the first jogging session (6:00 AM), breakfast (7:00 AM), lunch (12:30 PM), club activity training (4:30 PM–7:30 PM), second jogging session (7:40 PM), and dinner (8:30 PM). The meals were prepared by a nutritionist and dietitians, who provided three controlled meals daily. Energy intake was determined individually by multiplying 20 kcal by the ideal weight (height [m]^2^ × 22 [kg/m^2^; body mass index]), based on our previous research on dietary restrictions for athletes and overweight individuals during weight loss (reference energy intake range approximately 1100–1400 kcal/day) [7, 10] Participants in the WL group were informed about the potential reduction in energy balance during the intervention (approximately 1.5–2.0 kg of body fat assumed). The distribution of energy intake was set as follows: 150 kcal for breakfast, 350 kcal for lunch, and 600–900 kcal for dinner, with adjustments made for individual energy intake at dinner [7]. A sample of a daily meal plan has been described in our previous research [7]. As this study also provided nutritional education for athletes and active college students undergoing weight loss, the meals were designed to be easily obtainable, primarily from convenience stores. Considering the practical protein intake range of 1.2–1.7 g/kg for athletes and a ratio of 1.4–1.5 g/kg of body mass as the target for protein intake, we aimed to provide an appropriate protein intake for the participants to maintain muscle mass [8]. Water intake remained unrestricted, and participants were offered nonenergy-containing beverages and zero-calorie labeled jellies when experiencing hunger. Additionally, participants were instructed to refrain from consuming any meal apart from those provided. Further details regarding the meal restriction protocol have been described in our previous study [7, 8].

### 2.3. Muscle Functions

The participants performed hand grip strength, maximum leg extensor power, and isometric knee extension/flexion peak torque tests. Maximum voluntary handgrip strength was measured in the right and left hands using a digital handgrip dynamometer (Grip-D; Takei Kiki Kogyo Co., Ltd., Niigata, Japan) [12]. The grip width was adjusted using a screw to ensure that the proximal interphalangeal joint (second joint) was at a 90-degree angle. The participants positioned their feet shoulder-width apart and maintained an upright posture with their forearms extended away from the body. Measurements were conducted twice, alternating between the left and right sides, with participants instructed to exert maximum force. The highest recorded value for each side (left and right) was considered the representative value, and subsequently, the average value was calculated. The maximum leg extensor power was determined using an isokinetic dynamometer (Aneropress 3500; Combi, Tokyo, Japan). The participants were instructed to assume a deep-seated position within the chair device with both feet firmly placed on the footplate. Footplate positioning was meticulously adjusted to maintain a 90-degree angle at the knee joint while securing the legs with a belt. The load was calibrated to the body mass of the individual, and the maximum value was determined five times [13]. We used a versatile muscle function evaluation exercise apparatus to assess isometric knee extension/flexion peak torque (BIODEX System 3, Biodex Medical Systems, Shirley, NY, USA) [14, 15]. The participants were seated on a device chair, ensuring unrestricted knee movement, and secured with belts on the thigh, shoulder, and hip. Before primary measurements, one or two preliminary practice sessions were conducted. The measurements were conducted three times at 60°/s and 180°/s for each leg, with a 30 s intermission between trials. Participants were instructed to exert maximum effort during the measurements. Subsequently, the maximum value of peak torque (Nm) during knee joint flexion and extension was evaluated.

### 2.4. Jump Performance

Vertical jump measurements were used as jumping performance indicators. Markings were made on a stationary wall at regular intervals, and a high-speed video camera (Photorn Inc., Yamagata, Japan) was fixed at the recording position to capture jumps. Participants were instructed to maintain an upright posture, place their hands on their hips, and jump without running up. To record the vertex position, we ensured the participants wore swim caps during the jump, and the maximum vertex was evaluated using the recorded videos. The measurements consisted of two single jumps, and the average was assessed. After an adequate rest period, the participants performed continuous jumps for 1 min (jump/3 s, total 20 times/min) and were evaluated.

### 2.5. Statistical Analyses

The results are presented as the mean ± standard deviation. A two-way repeated-measures analysis of variance (ANOVA) was performed with group (WL vs. CON) and intervention (pre- and postintervention) as between-participant factors. Group differences were assessed using an independent-sample *t*-test. Changes in weight loss before and after treatment were compared between the groups using a paired *t*-test. Pearson's correlation coefficients were used to measure the association between vertical jump and body mass, FFM, muscle strength, and power. A power analysis was conducted to determine the requisite sample size for the two-way repeated-measures ANOVA. Using G∗Power software (version 3.1.9.6), we established the significance level (*α*) at 0.05 and the power (1 − *β*) at 0.95, with the effect size set at *f* = 0.4. The analysis indicated that a total sample size of 24 participants was necessary to detect significant effects. Statistical analyses were performed using RStudio software for Mac (RStudio version 1.3.959, 2009–2020 RStudio, Inc., Boston, MA, USA) and IBM SPSS 29.0 for Mac (IBM Corp., Armonk, NY, USA). For all analyses, statistical significance was set at a *p* value < 0.05.

## 3. Results

**Figure 1:**
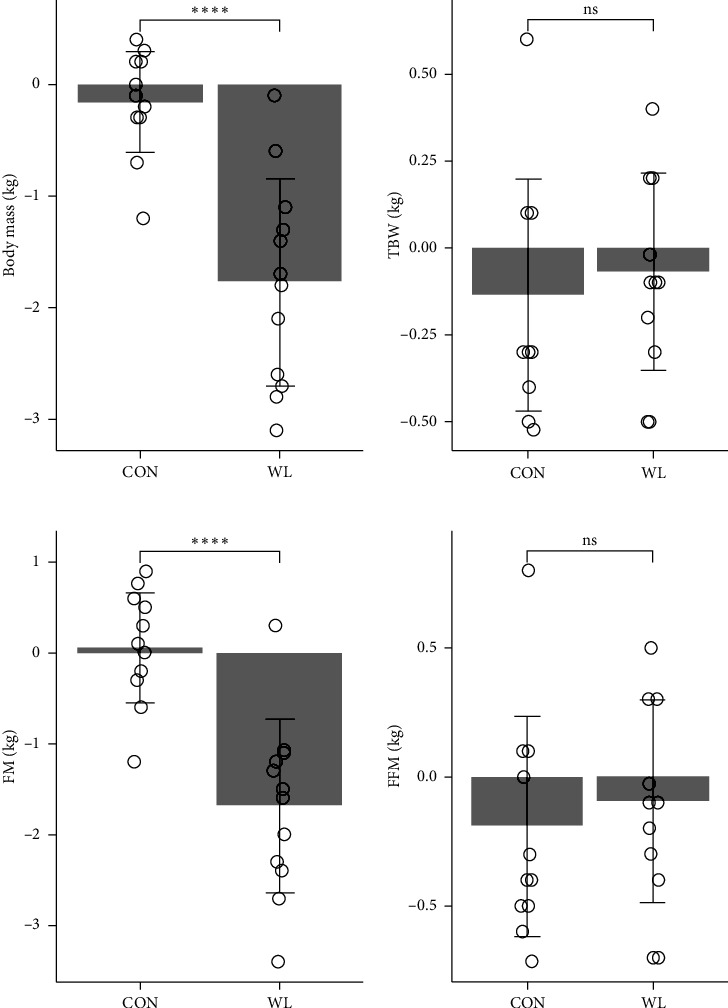
Changes in body mass and composition. Data are shown as mean ± SD. Each plot represents one individual. (CON: *n* = 12, WL: *n* = 12). CON: control, FFM: fat-free mass, FM: fat mass, SD: standard deviation, TBW: total body water, WL: weight loss. ⁣^∗∗∗^*p* < 0.01 between the groups.

The changes in body mass and composition are summarized in Table 1. We observed a significant interaction between group and weight loss for body mass (*F* = 29.375, *p* < 0.001, *η*_*p*_^2^ = 0.572) and FM (*F* = 24.793, *p* < 0.001, *η*_*p*_^2^ = 0.554). Both values significantly decreased in the WL group after the intervention, whereas no significant changes were observed in the CON group. A two-way ANOVA of TBW and FFM showed no interaction effect (TBW: *F* = 0.071, *p*=0.792, *η*_*p*_^2^ = 0.004; FFM: *F* = 0.093, *p*=0.764, *η*_*p*_^2^ = 0.005). Additionally, no significant main effects of the group were observed (TBW: *F* = 0.002, *p*=0.969, *η*_*p*_^2^ = 0.000; FFM: *F* = 0.002, *p*=0.966, *η*_*p*_^2^ = 0.000). We noted a significant difference in body mass and FM changes between the groups, although no significant differences were observed in the changes in TBW or FFM (Figure 1).

**Figure 2:**
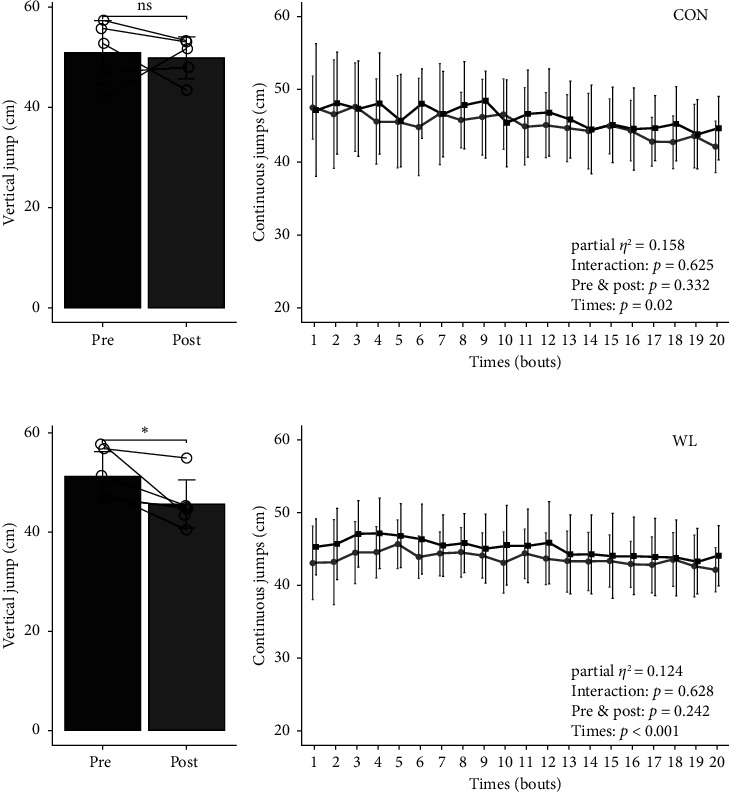
Counter movement jump and continuous jumps changes within individuals. Data are shown as mean ± SD. Each plot represents one individual. The black line plot (■) shows the precontinuous jumps and gray line plots (●) show postcontinuous jumps. (CON: *n* = 5, WL: *n* = 6), CON: control, FFM: fat-free mass, FM: fat mass, SD: standard deviation, TBW: total body water, WL: weight loss.

**Figure 3:**
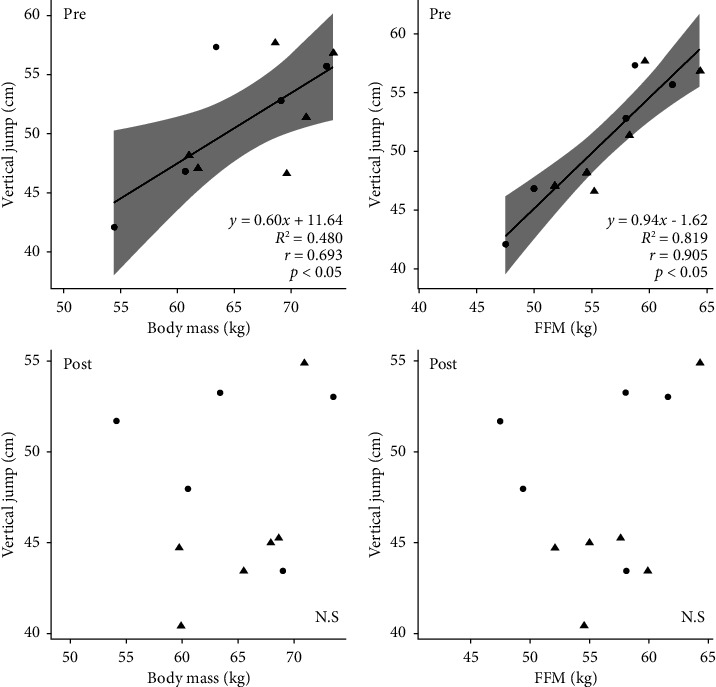
Relationship between vertical jump body mass and FFM of pre- and postmeasurements. Each plot shows the CON group (○) and WL group (△). (*n* = 11). CON: control, FFM: fat-free mass, WL: weight loss.

The strength test results, including effect sizes and *p* values, are listed in Table 2. We did not observe a significant interaction between group and weight loss in hand grip, knee extension test, peak knee extension, or flexion torque (60° and 180°).  Figure 2 shows the comparisons of single vertical and continuous jumps within individuals. The single vertical jumps were not significantly different pre- vs. postintervention in the WL group (50.9 ± 6.4 cm vs. 49.9 ± 4.2 cm, *p*=0.756, Cohen's *d* = 7.05). In addition, continuous jumps (20 repetitions in a minute) were not significantly different within individuals in each bout (pre- vs. postintervention). Single vertical jumps were significantly different pre- vs. postintervention in the WL group (51.3 ± 4.9 cm vs. 45.6 ± 4.9 cm, *p*=0.036, Cohen's *d* = 0.48). However, continuous jumps of 20 times were not significantly different within individuals in each bout (pre- vs. postintervention).  Figure 3 shows the pre- and postintervention relationships between single vertical jump, body mass, and FFM. There was a significant preintervention relationship between vertical jump and body mass (*r* = 0.693, *p* < 0.05) and FFM (*r* = 0.905, *p* < 0.05), although no postintervention relationship between vertical jump and body mass and FFM. There was no significant relationship between the changes in body mass, FFM, and vertical jumps before and after weight loss. In addition, the preintervention vertical jump correlated with the peak knee flexion torque at 60°/s and 180°/s before and after the intervention (60°/s: *r* = 0.730, 180°/s: *r* = 0.756, *p* < 0.05; 60°/s: *r* = 0.608, 180°/s: *r* = 0.746, *p* < 0.05, respectively). However, there was no significant relationship between changes in peak knee flexion torque and vertical jumps before and after weight loss.

## 4. Discussion

This study investigated the impact of a 1-week weight loss program without dehydration on the jumping performance and muscle power of volleyball players. The rationale behind this investigation stems from the importance of enhanced vertical jumping capability in volleyball, with a focus on maintaining FFM and losing FM to improve performance. The main findings indicated that although the 1-week weight loss program resulted in a significant decrease in FM and maintained FFM, jump performance did not improve. Notably, single jumps exhibited a decrease in height, whereas continuous-jump performance remained unaffected. Despite the maintenance of muscle power, the decrease in single-jump height contradicts the initial hypothesis, which anticipated improvements in jump performance following FM loss and FFM maintenance.

### 4.1. Body Composition and Weight Changes

The WL group demonstrated a significant decrease in body mass and FM following the 1-week intervention while maintaining FFM. Conversely, these changes were not observed in the CON group, emphasizing the specific effects of the weight-loss program. Similarly, the WL group showed no significant change in TBW compared to the CON group (Table 1). This finding is in line with previous findings conducted on university athletes, where an average energy deficit exceeding 2300 kcal/day was achieved through exercise and dietary restriction, resulting in reductions in body mass and FM while maintaining FFM over 1 week [8], as well as with our previous study targeting individuals of healthy weight, which achieved a reduction of 1.9 kg in fat mass through a deficit of 2311 kcal/day [7]. The current study also indicated a decrease in FM (1.7 kg), suggesting that the program could achieve an equivalent weight loss effect with a negative energy balance of 2257 kcal/day, calculated from body composition changes.

### 4.2. Muscle Functions

Although the weight-loss program significantly decreased FM, it did not significantly affect TBW or FFM. This was a positive finding, given that maintaining FFM is crucial for athletes' strength and power [16]. After the intervention, the strength and power tests, including handgrip, leg extension, and isometric knee extension/flexion peak torque, showed no significant differences between the groups, indicating that the weight loss program did not impair participants' muscle strength and power. Preserving these attributes is crucial for athletes to sustain peak performance during explosive movements such as jumps. Kraemer et al. [17] reported that wrestlers experienced a 6% decrease in body weight over 1 week, resulting in decreased torque values for knee flexion, knee extension velocity, and elbow extension velocity. Barley et al. [18] demonstrated differences in the ability to perform repetitive isometric strength tests after 3 h of dehydration (3%–4% body weight loss), indicating that dehydration leads to decreased muscle power. Although both previous studies mentioned a potential decrease in muscle power due to dehydration, our short-term weight loss protocol without dehydration likely resulted in no observed decline in muscle function. However, a study by Roklicer et al. [19] showed that 5% weight loss achieved after 1 week (4 days of specific vigorous training and 3 days of rapid weight loss) resulted in significant muscle damage in judo athletes. It should be noted that combat sports rely almost exclusively on dehydration to reduce weight rapidly [20], whereas in our case, the athletes were euhydrated.

### 4.3. Jump Performance

The analysis of vertical jumps yielded intriguing findings. Continuous vertical jumps performed 20 times did not exhibit significant differences within the WL group, suggesting that the 1-week intervention did not compromise immediate jumping performance. However, single jumps showed a notable decrease in height in the WL group. Although both FFM and TBW were maintained and muscle power was preserved, leading us to anticipate an improvement in jump performance, contrary to our hypothesis, single-jump performance decreased, whereas continuous-jump performance was sustained. Initially, the decline in single-jump performance was attributed to increased fatigue due to increased exercise volume. For instance, previous research has indicated that while there were no noticeable effects on jump performance in judo athletes before and after weight loss, a significant decline in vertical jump power was observed as the tournament progressed [17]. Acclimatization to short-term weight loss may also be an influencing factor. Volleyball players who are not accustomed to rapid weight loss might have experienced effects related to negative adaptation or mental aspects and body dexterity after weight reduction, despite the absence of dehydration, which could have hindered performance improvement [21, 22]. Additionally, we speculate that slow jogging to enhance energy expenditure may temporarily decrease the elasticity of the achilles tendon [23], thus potentially impeding the efficient utilization of elastic energy during jump height performance, given that the stretch-shortening cycle is slower than jumping actions. Therefore, new exercise interventions may be necessary to improve jump performance with increasing energy expenditure.

### 4.4. Practical Implications

The findings of this study suggest that a well-controlled, 1-week weight loss program focusing on lower body strength and optimal body composition can result in significant fat loss without compromising muscle functions crucial for sports performance. In addition to their usefulness in jumping, these research results may be applicable to other disciplines, such as judo and wrestling, which require maintenance of muscle function and reduction of FM. To enhance jumping capability, volleyball players and coaches may consider implementing weigth loss interventions for specific training and games. Additionally, this study emphasizes the importance of monitoring different aspects of jumping performance while considering variations in jump tasks and duration.

### 4.5. Limitations

This study has a few limitations. First, this study used basic vertical single and continuous jumps to evaluate performance. However, volleyball is a multifaceted sport that involves the use of a ball, thus fundamental and practical performance tests such as variations in jumping, agility, and cognitive functions, should be evaluated in future studies. Second, this study focused on an athletic discipline that is not accustomed to weight loss. Thus, further research exploring the psychological and skill-related facets of performance could offer a more comprehensive understanding of the impact of weight loss programs on overall volleyball performance.

## 5. Conclusions

We found that a 1-week weight loss program without dehydration did not affect FFM, hand grip, or lower limb muscle power of athletes, nor did it enhance jump performance. However, the program effectively reduced FM without negatively impacting muscle strength and power. Further research is needed to optimize such interventions to enhance jump performance and overall athletic ability.

## Figures and Tables

**Table 1:** Change in body mass and composition before and after intervention.

	Con (*n* = 12)		WL (*n* = 12)		Effect size	*p*
	Pre	Post	Pre	Post	Partial *η*^2^	*G* × *T*
Body mass (kg)	65.2 ± 5.7	65.1 ± 5.7	64.7 ± 6.9	62.9 ± 6.4⁣^∗^	0.572	<0.001
TBW (kg)	39.9 ± 3.1	39.8 ± 3.1	39.7 ± 5.1	39.7 ± 5.0	0.015	0.567
FM (kg)	10.7 ± 2.8	10.7 ± 2.9	10.4 ± 3.1	8.7 ± 3.3⁣^∗^	0.561	<0.001
FFM (kg)	54.6 ± 4.3	54.4 ± 4.2	54.3 ± 7.0	54.2 ± 6.8	0.018	0.537

*Note:* Values are presented as the mean ± SD. ⁣^∗^Significant difference (*p* < 0.05) pre- vs. postintervention in individuals.

Abbreviations: FFM, fat-free mass; FM, fat mass; TBW, total body water.

**Table 2:** Change in physical function before and after intervention.

	CON (*n* = 6)		WL (*n* = 12)		Effect size	*p*
	Pre	Post	Pre	Post	Partial *η*^2^	*G* × *T*
Hand grip (kg)	41 ± 8	43 ± 7	40 ± 8	39 ± 8	0.079	0.275
Maximum leg extensor power (watt)	1414 ± 194	1522 ± 508	1273 ± 285	1249 ± 344	0.040	0.444
*Peak knee extension torque*						
60°/s (Nm)	175 ± 48	185 ± 37	176 ± 29	173 ± 24	0.128	0.145
180°/s (Nm)	123 ± 22	128 ± 16	114 ± 28	107 ± 18	0.099	0.202
*Peak knee flexion torque*						
60°/s (Nm)	92 ± 18	102 ± 11	91 ± 23	103 ± 18	0.003	0.839
180°/s (Nm)	79 ± 14	91 ± 16	76 ± 25	83 ± 15	0.013	0.648

*Note:* Values are presented as the mean ± SD. ⁣^∗^Significant difference (*p* < 0.05) between pre- and postintervention within individuals.

Abbreviations: G, group (CON and WL); T, time (pre- and postintervention).

## Data Availability

The data that support the findings of this study are available from the corresponding author upon reasonable request.
